# Achieving Metabolic Flux Analysis for *S. cerevisiae* at a Genome-Scale: Challenges, Requirements, and Considerations

**DOI:** 10.3390/metabo5030521

**Published:** 2015-09-18

**Authors:** Saratram Gopalakrishnan, Costas D. Maranas

**Affiliations:** Department of Chemical Engineering, The Pennsylvania State University, University Park, PA, USA; E-Mail: sxg375@psu.edu

**Keywords:** genome-scale MFA, *S. cerevisiae*, challenges, requirements, considerations

## Abstract

Recent advances in 13C-Metabolic flux analysis (13C-MFA) have increased its capability to accurately resolve fluxes using a genome-scale model with narrow confidence intervals without pre-judging the activity or inactivity of alternate metabolic pathways. However, the necessary precautions, computational challenges, and minimum data requirements for successful analysis remain poorly established. This review aims to establish the necessary guidelines for performing 13C-MFA at the genome-scale for a compartmentalized eukaryotic system such as yeast in terms of model and data requirements, while addressing key issues such as statistical analysis and network complexity. We describe the various approaches used to simplify the genome-scale model in the absence of sufficient experimental flux measurements, the availability and generation of reaction atom mapping information, and the experimental flux and metabolite labeling distribution measurements to ensure statistical validity of the obtained flux distribution. Organism-specific challenges such as the impact of compartmentalization of metabolism, variability of biomass composition, and the cell-cycle dependence of metabolism are discussed. Identification of errors arising from incorrect gene annotation and suggested alternate routes using MFA are also highlighted.

## 1. Introduction

Metabolism in yeasts is primed for the maintenance of homeostasis while ensuring the ability to adapt to changing environmental conditions [1,2]. Unique metabolic characteristics have made yeasts key microbial producers in the fermentation industry [3], pharmaceutical industry [4,5], biofuel production [6,7], and bioremediation [8,9,10]. In particular, *Saccharomyces cerevisiae* has been extensively utilized for the production of isoprenoids, polyphenols, and recombinant eukaryotic proteins. Central metabolism in yeast is typically quantified using isotope tracer techniques [11], whereas peripheral metabolism is analyzed using FBA relying on linear and mixed integer programming approaches [12,13,14]. Available genome-scale metabolic (GSM) models are analyzed using metabolite balancing techniques such as FBA and FVA [15,16], and used to guide metabolic engineering [17] using strain design frameworks such as OptKnock [18]. GSM-aided metabolic engineering has guided the design of strains for improved production of compounds such as ethanol [17], succinate [19], vanillin [20], and sesquiterpenes [21]. In addition to this, GSMs for *S. cerevisiae* have been used to identify essential genes and lethal gene pairs [22,23], and as a platform to integrate high-throughput omics data [24,25,26]. On the other hand, isotope tracer techniques such as 13C-metabolic flux analysis (13C-MFA) [27] employ stable isotopes of carbon to estimate intracellular fluxes by minimizing the variance-weighted sum of square of deviation from experimentally observed labeling distribution of intracellular metabolites. The strength of this technique lies in its ability to resolve key metabolic branch points such as glycolysis/PPP [28] and fermentation/respiration [29] and reversible reactions by exploiting distinct pathway atom transitions. The scale of metabolic models utilized for 13C-MFA remain skeletal, only encompassing central metabolism due to computational challenges arising from structural identifiability of parameters limited by paucity of experimental data. Recent efforts have successfully accomplished genome-scale 13C-MFA in *E. coli* [30], highlighting the loss of information associated with the assumptions contained within core MFA models, the role of alternate metabolic routes, and a network-wide cofactor balance resolution, not achievable using a core model. This is of particular interest in yeast metabolism due to the prominent role of cellular redox state in metabolic shifts [31] and periodic metabolic cycles [32].

*Saccharomyces cerevisiae* is the most extensively studied species of yeast using 13C-MFA [33]. This technique has been utilized to characterize metabolic responses associated with catabolite repression [34] and oxygen availability [29,31], assess cell-cycle dependence of central metabolism [32], quantify the effect of gene knockouts [35], explore overproduction capabilities [36] and non-native substrate metabolism [37,38]. In all these cases, the mapping model contains lumped reactions from glycolysis, pentose phosphate pathway, TCA cycle, glyoxylate shunt, and ethanol production with very limited compartmentalization [39]. 13C-MFA has been employed to assess the three routes of glycine biosynthesis revealing enhanced glyoxylate shunt activity during growth on non-fermentable carbon sources [40,41]. The role of glucose repression during batch cultivation in breaking the TCA cycle and causing it to operate as two separate branches was also highlighted in the same study. Another study demonstrated flux re-routing towards ethanol production followed by drastic reduction in TCA flux and oxidative phosphorylation at oxygen levels less than 2.8% [31]. In a study using elutriated cells, changes in the glycolysis/PPP split ratio were observed and was attributed to the increased demand of reducing equivalents during certain phases of the cell-cycle [42]. Increased PPP flux following malic enzyme knockout has also been confirmed by altered labeling distribution in intracellular metabolites and amino acid fragments [35]. More recent studies have employed 13C-MFA to highlight a metabolic cycle existing between upper glycolysis and the pentose phosphate pathway in a strain engineered to grow on xylose [37]. Most of the MFA studies in yeast are aimed at estimating two key split ratios: glycolysis/PPP and respiration/fermentation. In all of these studies, the role of redox homeostasis is inferred based on flux re-distribution while no direct evidence from flux maps is available as the inclusion of cofactor balances is typically not included in yeast MFA models [43]. This is due to uncertainties due to compartmentalization and the presence of isozymes with different cofactor requirements [44,45]. Several studies have confirmed that mitochondrial kinases, which are beyond the purview of core MFA models, serve as major NADPH sources [46,47], thus warranting a re-examination of such hypotheses in the context of genome-scale models. On the other hand, the analysis of peripheral metabolism (soluble pool components, fatty acids, and other macromolecules) and production capabilities of secondary metabolites [48] is accomplished via FBA applied to a GSM model. 13C-MFA using genome-scale metabolic mapping (GSMM) models allows the incorporation of all metabolite balances without the pathway activity assumptions to provide an unbiased estimate of the flux solution space, which can be used to make high quality inferences about the metabolic state of the cell.

This review is aimed at outlining a workflow, key requirements, challenges, and considerations for successfully performing 13C-MFA at the genome-scale in *S. cerevisiae*. Three major requirements in terms of accuracy of model annotation, reaction atom mapping and sufficiency of metabolite labeling data are established. The sources of flux resolution loss arising from compartmentalization of metabolism and alternate routes, and additional data necessary to completely resolve such flux are discussed. Finally, approaches to address the computational challenges arising from increased network complexity and variability of the biomass equation arising from accumulation of macromolecules are proposed.

## 2. Requirements for Genome-Scale MFA

The primary requirements for performing MFA at the genome-scale are the availability of a well curated GSM model for *S. cerevisiae*, atom mapping data for every reaction within the GSM model, and intracellular metabolite labeling distribution data to resolve all fluxes within the GSM model. Despite various modifications to the consensus yeast model [23,49,50,51,52,53], its accuracy in predicting single gene lethality effects remains at only 29% for the most recently published Yeast 7 metabolic model [49]. This model was further updated to yield the current GSM model of *S. cerevisiae* containing 3494 reactions and 2223 metabolites [22] by adding 21 reactions, correcting 28 GPRs, and removing three incorrectly annotated reactions. This increased the specificity [23] of the *S. cerevisiae* GSM model from 28% to 35%. In contrast, the *E. coli* GSM model (*i*AF1260) has a specificity of 79% [23]. The lower specificity of the current *S. cerevisiae* model arises from its poorly characterized sterol and soluble pool biosynthetic pathways, and differences in biomass composition of mutants and wild-type [22]. However, metabolite flows through the well annotated central metabolism yields growth predictions for the current model [49,54] with an accuracy similar to that of the current *E. coli* model [55,56] confirming the applicability of 13C-MFA for reliable flux resolution in central metabolism. Reactions from the incompletely annotated pathways (fatty acid and cofactor biosynthetic pathways) alter the labeling distributions of measured metabolites by the production of small molecules such as CO_2_ and formate [30], which are subsequently re-incorporated into central metabolism, thereby causing errors in fluxes estimated using 13C-MFA. However, these errors are likely to be minimal due to the fact that FVA predicts very small flux through these pathways. Despite their minimal impact on central metabolic fluxes, the reliability of the estimated flux through these pathways remains low and thus, inferences must be treated with caution. Errors in the form of shifted or expanded flux ranges are likely to arise from incorrectly predicted gene lethality, 82% of which are associated with growth-coupled reactions contained within peripheral pathways. In order to reliably resolve energy metabolism, reconciliation of 21 prediction inaccuracies within oxidative phosphorylation is necessary. Only afterwards the corrected model can be employed for 13C-MFA after simplification by elimination of inactive reactions.

The reduction of this model can be accomplished by imposing fermentation data as mass balance constraints and eliminating all blocked reactions using FVA. A fermentation process involving *S. cerevisiae* is quantified in terms of growth rate, biomass and product (acetate and ethanol) yields on glucose. Respiratory quotient (CO_2_ evolved per mole oxygen consumed) is also routinely measured to constrain overall oxygen uptake. This restricts NADH re-oxidation and ATP synthesis via oxidative phosphorylation, thereby constraining network-wide redox metabolism. Incorporation of transcriptomic and proteomic data using frameworks such as binary ON/OFF type regulation [57] or R-GPR switches [58] may further reduce the number of active reactions and even improve gene knock-out predictions [59]. For example, the transcriptomic responses to the metabolic shift from respiration to fermentation have already been identified [60,61,62]. However, many reaction combinations forming thermodynamically infeasible and futile cycles may remain. It is important to note that the predicted labeling patterns of intracellular metabolites are insensitive to such reactions, making them structurally unidentifiable parameters. The final modification that needs to be made before the model can be utilized for 13C-MFA is the direction selection of reversible reactions whose exchange fluxes cannot be resolved by 13C-MFA. This can be accomplished using fermentation data-constrained FVA. In such cases either the forward or the reverse reaction can be eliminated from the model without impacting the optimal flux distribution and its confidence interval.

Atom mapping information for central metabolism remains conserved across all species and is largely readily available. Atom mapping for peripheral metabolism can be obtained from online databases such as MetaCyc [63], KEGG [64] and MetRxn [65]. In addition, *S. cerevisiae* contains yeast-specific pathways such as the α-aminoadipate pathway for lysine biosynthesis for which atom mapping has been established [66]. Characterization of promiscuity of enzyme activity has added novel metabolic reactions such as the riboneogenesis pathway [67] for which atom mapping remains poorly established. For such reactions, mapping algorithms based on graph theory are available [68]. In particular, the recent CLCA algorithm has been shown to be faster and more accurate in generating reaction atom maps in compared to previous algorithms due to the constraints imposed by chemical and stereo-chemical properties of reactions [69]. Complex chemical entities and incorrect determination of alternate reaction maps necessitate that the generated maps must be manually inspected. The atom mapping data generated using such algorithms is usually ordered based on SMILES notation [68] or graph invariance numbers [70], which is often very different from IUPAC numbering schemes. The limited availability of inter-nomenclature conversion tools further complicates the inspection and correction of data, often requiring additional visual support provided in MetaCyc [68] and MetRxn databases [65].

Labeling distribution data for MFA is typically obtained using GC-MS [40], LC-MS [36], or NMR [71]. The set of measured metabolite fragments predominantly consists of intracellular or proteinogenic amino acids. Only 12 amino acids can be analyzed using GC-MS due to contamination of data arising from co-elution and multiple overlapping fragments [72]. However, NMR allows for the analysis of all amino acids except cysteine, tryptophan, asparagine, and glutamine, which are either degraded or converted to other amino acids during sample preparation [73]. LC-MS allows the quantification of sugar phosphates from glycolysis and PPP, 3-phosphoglycerate, phosphoenol pyruvate, and pyruvate [28] in addition to all the amino acids quantifiable using GC-MS. The labeling distribution of carbons in ethanol [35] and the off gas CO_2_ [74] have also been utilized for 13C-MFA. A major challenge with analysis of metabolite labeling data is the existence of cellular compartments, which causes the observed labeling pattern to be an average labeling over all intracellular compartment pools of that metabolite. A possible way to address this challenge would be to measure compartment-specific pool sizes of metabolites. Separation of compartments for pool size measurements can be achieved using the recently proposed “lab on a chip” concept [75,76]. This approach has been demonstrated to be capable of cell lysis and organelle separation using low sample volumes. However it is limited by robustness and the inherent trade-off between ease of fabrication and organelle separation efficiency. As a result, its applicability has only been demonstrated as a “proof of concept”. In addition to this, *in vivo* NMR spectroscopy can be utilized to obtain compartment-specific pool sizes and labeling distributions directly for some metabolites based on differences in the microenvironment of various compartments [77]. This technique requires that the concentration of the metabolite to be detected must be greater than 2 μmol/g-wet cell weight and that the metabolite must have distinct ionization states in different intracellular compartments. These two criteria limit the number and nature of metabolites that can be analyzed using this technique. In particular, this method works well for amino acids and intermediates of amino acid biosynthesis such as homoserine. Additional labeling data obtained by MS measurement of compartment-specific peptides can also be utilized [78,79], but it suffers from information loss associated with peptide mass de-convolutions [80,81] and the inability to differentiate compartment-specific amino acid pools in rapid exchange. Analysis of labeling distribution of other macromolecule precursors such as fatty acids [82] and nucleotides [83] can be employed in 13C-MFA. Since a single set of measurements is insufficient to resolve all fluxes contained in a metabolic model, the common practice is to integrate multiple measurements obtained using various techniques [36] and even data sets obtained using different carbon tracers [84] to better resolve of fluxes. Optimal measurement sets identified using an algorithm such as OptMeas [85] can be used to guide labeling experiment design. However, it is likely that steady-state labeling distributions alone may be insufficient to resolve parallel pathways, in which case a non-stationary analysis may be necessary. Contraction of flux ranges obtained using the MFA procedure [86] can be achieved by penalizing deviations from experimentally observed extracellular flux measurements, which often fully close carbon balances. It may be worthwhile to note that a genome-scale model can take advantages of mass balance constraints of non-carbon metabolites as well unlike core models. In addition to this, the existence of topological features such as flux coupling further decreases the necessary data required for complete resolution of the model.

## 3. Scale-up Considerations and Loss of Resolution

**Figure 1:**
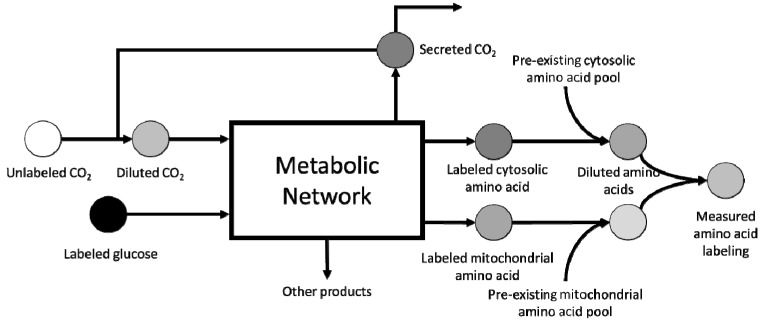
Corrections for metabolite pool dilution from various sources. Rapid exchange of intracellular and extracellular CO2 introduces unlabeled carbons into the metabolic network. Although its impact decreases with time, it still plays a significant role during mid-exponential phase [88]. Dilution of compartmental amino acid pools arises from pre-existing pools. The final measured labeling distribution is the average labeling across all compartmental pools.

Inability to completely resolve all fluxes included within the GSM model for *S. cerevisiae* occurs due to lack of probing techniques capable of obtaining compartment-specific labeling distribution of metabolites. Instead, existing procedures generate a pool-size-weighted average labeling distribution, which must be analyzed using corrections shown in Figure 1. This results in a degeneracy in compartment-specific labeling distributions, which is reflected in expanded flux ranges in the vicinity of that metabolite. Additional corrections to be considered during MFA of a eukaryote such as yeast include dilution by unlabeled CO_2_ from aeration and pre-existing metabolite pools. Furthermore, catabolism of storage compounds also adds unlabeled carbons, which may alter the estimated flux distributions if not properly accounted for. The longer doubling time of yeast results in slower biomass labeling, which in turn delays the attainment of isotopic steady-state. From the experimental perspective, the above described factors contribute to resolution loss, thereby affecting MFA flux inference. However, recent efforts have confirmed that loss of resolution can also arise from structural identifiability issues existing within the metabolic network [30]. In *S. cerevisiae*, loss of resolution can result from the existence of the methylglyoxal pathway and the γ-aminobutyrate pathway as alternate routes to lower glycolysis and TCA cycle, respectively, resulting in loss of resolution between these two pathways. A previous study has already attributed local flux range expansion to the presence of such alternate pathways in *E. coli*. In addition to this, intracellular compartmentalization of metabolism introduces metabolic cycles such as the malate shuttle [87], which are typically unresolvable using metabolite balancing techniques alone. The identification of additional metabolic loops arising from compartmentalization and their corresponding resolution criteria requires an in-depth analysis of the generated GSM mapping model of *S. cerevisiae*.

Another potential source of error is cell-cycle dependence of metabolic fluxes. It has already been demonstrated using synchronous cells that the glycolysis/PPP split ratio changes over a 24-h period [32], causing MFA to estimate an average flux distribution over one full cell cycle [89]. Metabolic responses to cell cycle phases can only be resolved by experiments with synchronous cells as sufficient tools are not available to analyze unsynchronized cells [80]. However, the flux distribution averaged over one cell doubling period can be reliably estimated by analyzing labeling distributions from a sufficiently large number of unsynchronized cells (~ 10^7^ cells). It must be noted that the reliability of the flux estimation procedure depends strongly on the attainment of isotopic steady-state and therefore, at least six doublings [45] must be allowed to minimize errors associated with isotopic non-stationary sampling. Presently, the only way to resolve cell-cycle dependence of metabolism is via synchronization of cells using available techniques [90], although, the use of cell cycle phase-specific peptides has been proposed [80].

## 4. Computational Challenges Associated with Model Scale-up

MFA at the genome-scale is performed by first decomposing the genome-scale atom mapping model into subnetworks using an appropriate decomposition algorithm such as the EMU algorithm and then estimating fluxes so as to minimize the deviation of predicted metabolite labeling patterns from experimental data. The fluxes are estimated by solving a non-linear least squares problem, which is then subjected to a goodness-of-fit analysis followed by confidence interval determination [30,86]. The immediate challenge associated with scale-up to a GSM model is the increase in the number of fluxes that need to be estimated resulting in a significant increase in computation time and memory. The current metabolic model of *S. cerevisiae* contain 3494 reactions and 2223 balanced metabolites [22], of which 856 are incapable of carrying flux during aerobic growth in minimal media containing glucose as the sole carbon source. Elimination of thermodynamically infeasible cycles further reduces the number of active reactions to 2227. A similar reduction has been observed with the *E. coli* model in which case the number of active reactions was reduced to 697 from 2383 using a flux variability analysis based on growth conditions [30]. In contrast, the corresponding core models for *S. cerevisiae* and *E. coli* contain 32 [39] and 75 [91] reactions, respectively. The poor scalability of the existing methods results in a need for fast and memory efficient algorithms for flux and range estimation using a GSM model. The MFA procedure estimates fluxes by solving a non-linear least-squares problem which minimizes the deviation of predicted metabolite labeling patterns from experimentally observed data [86] using local minimization algorithms. Free fluxes [92] are reported as the solution to the NLP, which are related to the fluxes in the metabolic model by means of the null space matrix of the S-matrix [93]. Presently, there are no algorithms available to accelerate the flux estimation process using a GSM model, although, network topological features such as flux coupling (either to biomass production or an extracellular flux measurement) [18] have been exploited to accelerate the determination of confidence intervals for all reactions contained within the GSM model. The prediction of metabolite labeling patterns for a given flux distribution is facilitated by decomposition of the model based on available experimental data using a variety of algorithms [91,92,94], of which, the EMU method identifies the largest sub-network necessary to simulate a tracer experiment. Interestingly, the EMU model only accounts for a fraction of all reactions contained within the GSM model, as a result of which, the number of free fluxes in the GSM model obtained based on the null space of the S-matrix is over-estimated, resulting in a larger non-linear problem with structural identifiability issues [95] and lack of statistical significance. These challenges can be addressed by identifying free fluxes corresponding to the EMU model, and not the entire metabolic model. For example, in *E. coli*, this transformation reduces the number of free fluxes from 250 to 99, thereby ensuring statistical significance of the obtained flux distribution [30]. The direct analysis of the EMU model has been proposed [30], however, the computational tools for this purpose are not yet available. Another suggested approach is the reduction of the GSM model for MFA to the size of a typical core model without any loss of information via assumptions with the aim of greatly reducing computational time. Available algorithms such as pFBA can be employed for simplification of complex pathways to facilitate model reduction [96]. Reduction in computational time is expected to greatly improve the utility of genome-scale MFA and make it a practical tool in the analysis of co-cultures [97] and isotopic non-stationary systems [98].

Growth-coupling as a means to speed up confidence interval estimation must be used with caution in an organism such as *S. cerevisiae*. While the biomass composition of a prokaryote such as *E. coli* remains relatively constant [99], much variation of macromolecule content associated with changing environmental conditions has been reported in *S. cerevisiae* [100,101]. In particular, a three-fold reduction in protein and nucleotide content and a two-fold increase in carbohydrate content in response to nitrogen starvation has been demonstrated. Furthermore, it has also been shown that this variability in biomass composition also affects the accuracy of prediction of knockouts in the current yeast model [22], thus, making it necessary to quantify the biomass composition for every growth condition analyzed [102] using available techniques [103]. While such variations directly affect growth-coupled reactions, the sensitivity of central metabolism and reactions outside the purview of EMU models to such perturbations remains to be seen.

## 5. Conclusions

In this review, we highlighted the various requirements, challenges, and considerations for achieving genome-scale flux resolution using 13C-MFA at isotopic and metabolic steady-state. While skeletal central metabolic models continue to be the norm, genome-scale MFA holds the potential to validate the various hypotheses proposed by previous analyses [29,31,35,37,38]. The current genome-scale model of *Saccharomyces cerevisiae* has a much lower prediction specificity compared to *E. coli* [23,49], which may have an adverse impact on the inference of fluxes through poorly annotated pathways. The generation and curation of a genome-scale atom mapping model is also necessary for 13C-MFA, for which tools are already available [68,69]. Spectroscopic tools for analysis of labeling distributions have already been well established and routinely utilized for MFA [28,29,31,104]. This allows for easy integration of complementary labeling data obtained from GC-MS, LC-MS and NMR [105] for better flux resolution using MFA. Resolution loss associated with using a more complex model remains to be quantified. MFA using a GSM model could indirectly help identify incorrectly annotated reactions and help improve the prediction quality of the current yeast metabolic model. Successful genome-scale MFA currently requires more efficient algorithms for flux estimation owing to the poor scalability of existing methods. In particular, approaches using minimum number of variables and memory, while improving convergence to the true global minimum have to be developed. The design of model simplification algorithms holds the promise of greatly reducing computational complexity and time requirements, thereby expanding the scope and application of 13C-MFA.
